# Fraternal Birth Order Effect and Other Sibship Effects in Bisexual and Asexual Men and Women

**DOI:** 10.1007/s10508-026-03536-9

**Published:** 2026-09-22

**Authors:** Jakub Fořt

**Affiliations:** 1 Department of Zoology, Faculty of Science, Charles University, Viničná 7, 128 00 Prague, Czechia; 2 Center for Sexual Health and Interventions, National Institute of Mental Health, Klecany, Czechia; 3 Department of Psychology and Life Sciences, Faculty of Humanities, Charles University, Prague, Czechia

**Keywords:** Sexual orientation, Sibship size, Homosexuality, Asexuality, Birth order, Maternal immune hypothesis

## Abstract

Previous research has shown that homosexual men and women tend to have more older brothers than their heterosexual counterparts—this is known as the fraternal birth order effect (FBOE) and is usually considered a marker for biological development of sexual orientation. Recent studies suggest this effect might also apply to bisexual and asexual individuals who have been overlooked by previous scholarship. In this study, I explored associations of the FBOE and other sibship effects with minority sexual orientation in an online collected sample of heterosexual (*n* = 1174), bisexual (*n* = 432), and asexual (*n* = 90) men and women from Czechia and Slovakia. Results of the binary logistic regression indicated the FBOE in bisexual men (*OR* = 1.66) but not in other sexual minority groups. There were no significant differences in other sibship characteristics between sexual minority and heterosexual individuals, although other non-heterosexual study groups showed a non-significant FBOE of a magnitude similar to bisexual men. This study contributes to the limited literature on understudied bisexual and asexual populations and indicates that male bisexuality might share a common biodevelopmental origin with male and possibly also female homosexuality.

**Supplementary Information:**

The online version contains supplementary material available at https://doi.org/10.1007/s10508-026-03536-9.

## Introduction

Previous research has identified several factors that are associated with non-heterosexual orientation, focusing on both psychological differences among people of various sexual orientations (e.g., personality, mental health, or cognitive abilities; Bogaert et al., 2018a; Feinstein et al., 2023; Semenyna et al., 2025; Xu et al., 2020) as well as putative markers for biological influences on the development of sexual orientation (e.g., Ablaza et al., 2022; Aguilar, 2023; Ganna et al., 2019). In this article, I will concentrate on the so-called fraternal birth order effect (FBOE), a well-known preponderance of older brothers among gay men, which is possibly present also among other sexual minority groups. In our previous article (Fořt et al., 2024), we already presented results on the FBOE and other sibship effects in homosexual men and women. This article concerns additional analyses of bisexual and asexual men and women from the same dataset and is an extension to our previous work.

### Sibship Effects and Sexual Orientation

In comparison with straight men, gay men have a preponderance of older brothers, which is commonly referred to as the fraternal birth order effect, or the FBOE. This effect was demonstrated in several individual studies collected by various research teams in various geographic areas (e.g., Apostolou, 2020; Blanchard & Bogaert, 1996; Fořt et al., 2024; Kabátek & Blanchard, 2025; Sadr-Bazzaz & Vasey, 2025), including a very large dataset from a Dutch census (Ablaza et al., 2022), and was corroborated by meta-analyses (Blanchard, 2018a; Blanchard et al., 2021). As it seems, the FBOE concerns only maternal (i.e., sharing the same biological mother with the focal participant) and not non-maternal or non-biological older brothers (Bogaert, 2006; Fořt et al., 2024). It is therefore usually explained on a purely biological basis by the maternal immune hypothesis (MIH; Blanchard & Klassen, 1997). According to the MIH, the mother’s immune system is activated by foreign male antigens originating in the male fetuses during pregnancy. The mother develops antibodies against these antigens that should be able to cross the placenta during subsequent pregnancies and alter the unborn individuals’ development in a way that they become same-sex attracted adults. To this day, the MIH has been directly supported by one laboratory study (Bogaert et al., 2018b), which found higher blood levels of antibodies against the male-specific protein neuroligin 4 Y-linked (NLGN4Y) in mothers of adult gay men compared to mothers of straight men. As such, the FBOE is usually considered one of the putative markers of biological influence on sexual orientation (Swift-Gallant et al., 2019; VanderLaan et al., 2023). For detailed information on the MIH and its recent developments, see Blanchard and Klassen (1997), Bogaert et al., (2018b), Blanchard and Skorska (2022), and Kabátek and Blanchard (2025).

An alternative explanation for the FBOE, not mutually exclusive with the MIH, is the fetal microchimerism (Haig, 2014; see also Bogaert et al., 2018b). Fetal cells can cross the placenta, engraft in maternal tissues, and influence maternal physiology (Cómitre-Mariano et al., 2022). These cells can be detected for several decades postpartum in the maternal blood (Bianchi et al., 1996) and could, hypothetically, be responsible or co-responsible for the development of sexual orientation of the mother’s subsequent offspring. These cells could also contribute to continuous production of antibodies that affect the fetal development as the MIH suggests.

Apart from the FBOE in gay men, there is now evidence for other sibship effects in gay men and for their presence in men of other sexual orientations and in non-heterosexual women. Some studies recorded the sororal birth order effect (SBOE; e.g., Ablaza et al., 2022; Kabátek & Blanchard, 2025; Sadr-Bazzaz & Vasey, 2025; Zdaniuk et al., 2025) for gay men, meaning they have more older sisters than straight men. The relative difference in magnitude between the FBOE and the SBOE is theoretically important for the MIH. Briefly, if the SBOE is weaker than the FBOE, it could be explained by the association between liveborn female offspring and miscarried male embryos/fetuses (Blanchard & Lippa, 2021), i.e., just as another manifestation of the mechanism behind the FBOE. Alternatively, older sisters could influence their younger siblings’ sexual orientation in their own right—equally strong SBOE and the FBOE could mean autosomal antigens are probably involved in the immune reaction (original version of the MIH relied solely on the involvement of male-specific, Y-linked antigens; for details, see Kabátek & Blanchard, 2025). The difference between the FBOE and the SBOE can be formally computed using the so-called fraternal-sororal effect differential (FSED; Blanchard, 2022).

The FBOE was recorded in homosexual women and inspected in bisexual and asexual men and women in several studies during recent years. First, the FBOE in lesbian women was demonstrated in individual samples (Blanchard, 2022; Fořt et al., 2024; Kabátek & Blanchard, 2025), including the aforementioned Dutch census dataset (Ablaza et al., 2022). Some studies did not record the effect (Sadr-Bazzaz & Vasey, 2025; Zdaniuk et al., 2025), including a relatively recent meta-analysis (Vilsmeier et al., 2023). However, the most recent articles indicative of the FBOE in lesbian women were not included in this meta-analysis (Ablaza et al., 2022; Blanchard, 2022; Fořt et al., 2024; Kabátek & Blanchard, 2025). Some authors (Blanchard, 2022; Kabátek & Blanchard, 2025) argue that the FBOE in lesbian women could also be explained on an immunological basis—e.g., the immune reaction predicted by the MIH for male homosexuality generalizes and the antibodies also interact with proteins present in female fetuses (for further elaboration and alternative explanations, see Kabátek & Blanchard, 2025).

Only a handful of studies investigated sibship effects in bisexual and asexual individuals. Some demonstrated the FBOE in samples of bisexual men (Blanchard, 2022; Freudenfeld et al., 2024; Sadr-Bazzaz & Vasey, 2025; but see Apostolou, 2020; Kabátek & Blanchard, 2025; Zdaniuk et al., 2025). So far, there are only null results regarding the presence of the FBOE in bisexual women (Apostolou, 2020; Blanchard, 2022; Freudenfeld et al., 2024; Kabátek & Blanchard, 2025; Sadr-Bazzaz & Vasey, 2025; Zdaniuk et al., 2025). Despite one earlier report indicating asexual men have more older brothers than straight men (Yule et al., 2014), more recent studies did not testify for the FBOE in asexual men (Freudenfeld et al., 2024; Kabátek & Blanchard, 2025; Zdaniuk et al., 2025). No significant FBOE was found in asexual women when compared to heterosexual women (Freudenfeld et al., 2024; Kabátek & Blanchard, 2025; Yule et al., 2014; Zdaniuk et al., 2025).

There is another sibship effect associated with sexual orientation—the so-called female fecundity effect (FFE) is the notion that mothers of gay men have more offspring than mothers of straight men and was documented by some previous studies (e.g., Camperio Ciani et al., 2004; Iemmola & Camperio Ciani, 2009; Semenyna et al., 2017). This effect may interfere with ascertaining the FBOE and the SBOE, as having a larger sibship size automatically entails a higher probability of having more older brothers and/or older sisters and vice versa (Khovanova, 2020). More recent studies that used methods specifically devised to disentangle the FFE from the FBOE and the SBOE either did not show significant differences in mother’s fecundity between gay and straight men (Semenyna et al., 2023) or demonstrated lower sibship sizes for gay men’s mothers (Ablaza et al., 2022; Blanchard, 2022). These studies similarly showed either lower sibship sizes of homosexual women compared to heterosexual women (Ablaza et al., 2022; Blanchard, 2022) or no differences (Kabátek & Blanchard, 2025; Sadr-Bazzaz & Vasey, 2025; Zdaniuk et al., 2025). Most studies did not show any difference in sibship size between bisexual and heterosexual men (Blanchard, 2022; Kabátek & Blanchard, 2025; Sadr-Bazzaz & Vasey, 2025, but see Zdaniuk et al., 2025) and, similarly, most studies did not show a difference for bisexual and heterosexual women (Kabátek & Blanchard, 2025; Sadr-Bazzaz & Vasey, 2025; Zdaniuk et al., 2025, but see Blanchard, 2022). As far as I know, the only two studies that collected complete sibling data on asexual individuals gained conflicting results—Kabátek and Blanchard (2025) recorded smaller sibship sizes for both asexual men and women, while Zdaniuk et al. (2025) recorded larger sibship sizes for asexual men and no difference for asexual women. Further studies are, therefore, needed to determine the relationship between asexuality and sibship size.

The FBOE was originally considered a correlate of male homosexuality only. I suppose initial null findings in female samples (Blanchard, 1997; Bogaert, 1997) might have hindered the FBOE research in lesbian women, and the dearth of studies into other sexual orientations (bisexual and asexual) prevented the development of a general theoretical background of the biological basis of non-heterosexuality (not only homosexuality) in humans (not only men). Some previous studies amalgamated bisexual and homosexual participants into a common non-heterosexual sample (e.g., Skorska & Bogaert, 2020; Xu & Zheng, 2017; Yule et al., 2014). This may be one cause for the relative lack of studies on bisexual participants, together with the possibility that previous samples included too few bisexual participants to make a separate study group (Blanchard, 2023). In this article, my aim is to contribute to the recent debate (see Blanchard, 2023) on the FBOE and other sibship effects in previously understudied sexual orientations—bisexual and asexual men and women from Czechia and Slovakia.

## Method

In this section, I will describe the methods relevant to this research; however, additional information can be found in a previous article (Fořt et al., 2024).

### Participants

The data were collected between June 2020 and March 2021 online via Qualtrics. A total of 3963 respondents started the survey session. The following exclusion criteria are stated in a non-overlapping way, i.e., if an excluded participant is already counted in a preceding criterion, they are not counted in any of the subsequent criteria, even if they would formally meet the criteria. I excluded (1) participants who did not indicate their sex (*n* = 243) or indicated their sex as “other” (*n* = 50). From the remainder, respondents were excluded if they (2) were less than 18 years old (*n* = 38), more than 79 years old (*n* = 8), or did not indicate their age (*n* = 3). Additional participants were excluded if they (3) reported being adopted or were unsure about it (*n* = 56), (4) considered themselves transgender (*n* = 114), or (5) were classified as homosexual (*n* = 963). In case the participants did not consider themselves asexual, they were excluded if they (6) failed to fill in all five items to classify their sexual orientation (*n* = 760; see Measures of this article and Supplementary Figure S1 in Fořt et al., 2024). Additionally, participants were excluded if they (7) were unsure about the exact number in any category of their siblings—full siblings, paternal or maternal half-siblings, or step-siblings (*n* = 21). Finally, (8) participants (*n* = 11) who reported: having more than nine children, having more than eight full siblings, and/or their mother or father having more than nine children, including the participant, were excluded—having such a high number of relatives was considered unlikely given Czech demography (Czech Statistical Office, 2021). The final male sample comprised 843 heterosexual, 184 bisexual, and 58 asexual men. The final female sample comprised 331 heterosexual, 248 bisexual, and 32 asexual women.

### Procedure

The online questionnaire was distributed in Czech to both Czech and Slovak participants—Slovaks usually understand Czech perfectly and vice versa. The questionnaire included a brief introduction explaining the general purpose of the survey (titled “Relationship among sexual behavior, orientation, and biodemographic factors”) and an informed consent. The survey employed a targeted sampling method also employed elsewhere (Camperio Ciani et al., 2004). Various LGBT+targeted web and social media pages and influencers were asked to disseminate the survey. After that, web and social media pages and influencers that were not LGBT+targeted but resembled the previously contacted ones in their spheres of interest were also asked to disseminate the survey. This was done to minimize any potential differences between non-heterosexual and heterosexual participants with respect to their socioeconomic and cultural backgrounds. Finally, we employed a paid promotion on Facebook. No financial incentive was offered to the participants. For more details, see Fořt et al. (2024). Although the original research was primarily aimed at sampling homosexual and heterosexual participants, we were also able to collect data from participants of other sexual orientations.

### Measures

Here, I state only the variables that are relevant for this research. Demographic information included age, sex (“male,” “female,” and “other”), number of own biological daughters and sons, number of full siblings, maternal and paternal half-siblings, and step-siblings (older and younger brothers and sisters, each category contained “I do not know” option), number of siblings from a potential common multiple birth, importance of religious faith (from 0 meaning “not important” to 100 meaning “very important”), highest education attained, population size of the childhood place of residence, population size of the current place of residence, an item asking whether the participant was adopted: “Were you adopted?” (“yes,” “no,” “I am not sure”), and mother’s age. Individual levels of ordinal variables are stated in Table 1.

**Table 1:** Descriptive statistics of the sample

	Men			Women		
	Heterosexual (*n* = 843)	Bisexual (*n* = 184)	Asexual (*n* = 58)	Heterosexual (*n* = 331)	Bisexual (*n* = 248)	Asexual (*n* = 32)
Age, *M* (*SD*), n.s./*	32.89 (11.95)a	33.51 (13.29)a	29.72 (10.48)a	31.77 (9.78)a	26.40 (6.20)b	30.09 (10.58)ab
< Missing >	0	0	0	0	0	0
Mother’s age at participant’s birth, *M* (*SD*), n.s./n.s	27.17 (5.06)a	26.72 (5.22)a	26.65 (4.65)a	26.93 (4.93)a	26.39 (5.33)a	26.46 (5.95)a
< Missing >	234	54	15	42	32	6
Importance of religious faith, *M* (*SD*), */*	30.93 (33.84)a	23.75 (28.99)b	26.94 (30.70)ab	26.93 (30.87)a	17.15 (23.91)b	11.19 (18.65)b
< Missing >	92	27	11	29	34	1
Number of biological sons, *M* (*SD*), */*	0.37 (0.73)a	0.23 (0.52)b	0.07 (0.32)c	0.37 (0.71)a	0.14 (0.40)b	0.19 (0.47)ab
< Missing >	0	0	0	0	0	0
Number of biological daughters, *M* (*SD*), */*	0.35 (0.70)a	0.28 (0.67)ab	0.14 (0.58)b	0.37 (0.70)a	0.13 (0.46)b	0.19 (0.54)ab
< Missing >	0	0	0	0	0	0
Education, n (%), */*						
Primary	33 (3.9)	11 (6.0)	6 (10.3)	5 (1.5)	13 (5.2)	2 (6.3)
Upper secondary (vocational)	34 (4.0)	19 (10.3)	7 (12.1)	7 (2.1)	11 (4.4)	6 (18.8)
Upper secondary (high school graduation)	348 (41.3)	83 (45.1)	26 (44.8)	122 (36.9)	126 (50.8)	17 (53.1)
Tertiary (bachelor’s degree or equivalent)	121 (14.4)	21 (11.4)	9 (15.5)	77 (23.3)	47 (19.0)	1 (3.1)
Tertiary (master’s degree or equivalent)	247 (29.3)	37 (20.1)	9 (15.5)	102 (30.8)	47 (19.0)	4 (12.5)
Tertiary (doctorate or equivalent)	55 (6.5)	13 (7.1)	1 (1.7)	17 (5.1)	3 (1.2)	2 (6.3)
< Missing >	5 (0.6)	0	0	1 (0.3)	1 (0.4)	0
Population size of the childhood place of residence, n (%), */n.s						
< 1,000	83 (9.8)	30 (16.3)	5 (8.6)	39 (11.8)	41 (16.5)	5 (15.6)
1,000–5,000	151 (17.9)	38 (20.7)	7 (12.1)	53 (16.0)	46 (18.5)	4 (12.5)
5,000–50,000	247 (29.3)	56 (30.4)	26 (44.8)	96 (29.0)	68 (27.4)	11 (34.4)
50,000–500,000	234 (27.8)	38 (20.7)	16 (27.6)	73 (22.1)	52 (21.0)	7 (21.9)
> 500,000	125 (14.8)	21 (11.4)	4 (6.9)	68 (20.5)	41 (16.5)	5 (15.6)
< Missing >	3 (0.4)	1 (0.5)	0	2 (0.6)	0	0
*Population size of the current place of residence, n (%), n.s./n.s*						
< 1000	72 (8.5)	19 (10.3)	6 (10.3)	30 (9.1)	15 (6.0)	4 (12.5)
1000–5000	108 (12.8)	22 (12.0)	6 (10.3)	24 (7.3)	22 (8.9)	2 (6.3)
5000–50,000	161 (19.1)	48 (26.1)	17 (29.3)	61 (18.4)	41 (16.5)	8 (25.0)
50,000–500,000	258 (30.6)	45 (24.5)	20 (34.5)	77 (23.3)	68 (27.4)	8 (25.0)
> 500,000	224 (26.6)	46 (25.0)	9 (15.5)	127 (38.4)	94 (37.9)	9 (28.1)
< Missing >	20 (2.4)	4 (2.2)	0	12 (3.6)	8 (3.2)	1 (3.1)

Participants with a missing value were excluded from a relevant statistical test. Asterisks indicate a significant difference at *p* < .05 for men (before the slash) and for women (after the slash) based on chi-square test for categorical variables and one-way ANOVAs for quantitative variables (Welch’s if significant inequality of variances was indicated by Levene’s test, Fisher's in the case of equal variances). Means not sharing a common letter are different at *p* < .05. Complete test results are reported in the Supplementary Material

Sexual orientation was assessed using five Kinsey-like items regarding sexual self-identification, fantasies, desire, and the sex of sexual partners during the past year and the past five years (all 7-point scales, see Figure S1 in Fořt et al., 2024, for the exact wording of each item). Additionally, participants were asked: “Do you consider yourself asexual?” (“yes,” “no”) and: “Do you consider yourself a transgender person?” (“yes,” “no,” “I am not sure”).

### Data Preparation and Analyses

Data were prepared in Excel and analyzed in Jamovi (The Jamovi Project, 2024). For each participant, I computed a mean score of the five Kinsey-like items. Participants with a mean score 0–1 (inclusive) that did not indicate they were asexual were considered heterosexual. Participants with a mean score 1–5 (exclusive) that were not asexual were considered bisexual, and those with a mean score 5–6 (inclusive) that were not asexual were considered homosexual and excluded from the study. Participants were considered asexual based only on their self-identification. Some of these asexual participants did not answer all five Kinsey-like items (but many of them did, including items regarding the sex of their previous sexual partners), and I should explicitly note that based on their mean score on these items, they could have been considered straight, gay, lesbian, or bisexual.^1^

Participant’s sibship size was calculated as a sum of all maternal siblings (full siblings and maternal half-siblings, not including the participant and their siblings from a potential common multiple birth). All other relevant sibling variables (e.g., older brothers, older siblings) were calculated as a sum of full siblings and maternal half-siblings. Mother’s age at participant’s birth was calculated by subtracting the participant's age from the mother’s age; missing values were substituted with arithmetic means separately by group (heterosexual, bisexual, and asexual men and women).

Recently, there has been development in the analytical approaches to study sibship effects on sexual orientation. Historically, studies usually utilized binary logistic regression with sexual orientation as a dependent variable and four classes of siblings (older and younger brothers and sisters) as separate predictors. However, this conventional parameterization of logistic regression has been criticized for not being able to disentangle the FBOE from the FFE and the SBOE (for details, see Ablaza et al., 2022; Khovanova, 2020; Raymond et al., 2025). Analytically more elaborate parameterizations that aimed to resolve this issue have been proposed (Ablaza et al., 2022; Blanchard, 2022; Zdaniuk et al., 2025) and comprehensively explicated in a recent methodological article by Blanchard et al. (2025). My primary aim was to perform analyses that adhere to current developments in the field while also being comparable with results presented in our previous publication (Fořt et al., 2024). Therefore, to compare sexual orientation groups (asexual and heterosexual, and bisexual and heterosexual), I specified two logistic regression models with different parameterizations: the one described in Ablaza et al. (2022) and the second resembling the parameterization described in Blanchard (2022). The first model (hereinafter referred to as the “baseline parameterization”) contains the following predictors: (a) sibship size, (b) number of older siblings, (c) number of older brothers, and (d) number of younger brothers. The second model (hereinafter referred to as the “complementary parameterization”) contains the predictors: (1) sibship size, (2) number of older siblings, (3) number of older sisters, and (4) number of younger sisters. In all models, participant’s age and mother’s age at participant’s birth were included as covariates to control for birth cohort effects, as was advocated in Ablaza et al. (2022). We also controlled for these two covariates in our previous article (Fořt et al., 2024). Both parameterizations and the meaning and interpretation of their theoretically relevant predictors (not including the two covariates) are summarized in Table 2. G*power software, version 3.1.9.7 (Faul et al., 2007), was used to calculate post-hoc achieved power of selected associations in a binary logistic regression. A Poisson distribution of the independent variable was chosen, with a lambda set at 0.5, a two-tailed alpha at 0.05, and R^2^ of other predictors at 0.4. For each inspected association, I entered the total sample size, the probability of sexual minority presence under the null hypothesis, and the odds ratio that were actually achieved.

**Table 2:** Two differently parameterized logistic regression models and interpretation of their coefficients

Predictor	Coefficient	Interpretation	Theoretical meaning	
*Baseline parameterization*				
Sibship size	β_1_	One younger sister is added to sibship	FFE-ys	
Number of older siblings	β_2_	One older sister replaces one younger sister	SBOE	
Number of older brothers	β_3_	One older brother replaces one older sister	FSED	
Number of younger brothers	β_4_	One younger brother replaces one younger sister		
*Complementary parameterization*				
Sibship size	δ_1_	One younger brother is added to sibship	FFE-yb	
Number of older siblings	δ_2_	One older brother replaces one younger brother	FBOE	
Number of older sisters	δ_3_	One older sister replaces one older brother		
Number of younger sisters	δ_4_	One younger sister replaces one younger brother		

FFE-ys stands for the female fecundity effect based on adding an extra younger sister to the sibship, FFE-yb stands for the female fecundity effect based on adding an extra younger brother to the sibship, SBOE for the sororal birth order effect, FSED for the fraternal-sororal effect differential, and FBOE for the fraternal birth order effect

Both these parameterizations were performed in our previous article (Fořt et al., 2024). To some extent, they correspond to simplified parameterizations used in Blanchard (2022) and Kabátek and Blanchard (2025), with an important difference: the inclusion of the fourth predictor, i.e., the number of younger brothers or younger sisters. Although I agree with Blanchard’s (2022, p. 685) statement that the “three-predictor model eliminates the parameter of younger siblings’ sex, which is unimportant anyhow, and it also improves the assessment of sibship size,” I am mainly driven by the desire for comparability between models used here and those in our previous article. Therefore, I hold onto four-predictor models that are presented here. Nonetheless, analyses adhering to the most recent developments of the logistic regression parameterization (see Blanchard et al., 2025 and Zdaniuk et al., 2025) that do not contain the fourth predictor of younger brothers or younger sisters, respectively, are reported in supplementary Tables S1–S8. After applying these additional analyses, the results (in terms of any theoretically relevant predictor, see Table 2, crossing the significance threshold) did not change in any respect.

I note that including the number of younger brothers and younger sisters into the baseline and complementary parameterization, respectively, also entails an implication of obtaining two different FFE coefficients with different interpretations—a sibship-size effect based on adding an extra younger sister (FFE-ys) in the former case, or an extra younger brother (FFE-yb) in the latter case, to the sibship.

As the concurrent utilization of the baseline and the complementary parameterization poses a clear advantage (for summary, see Blanchard et al., 2025) over the older conventional parameterization (described above and used, e.g., in Blanchard & Boagert, 1996, and many other studies), I decided to retain only these two parameterizations here, in contrast to our previous article, where we also used the conventional parameterization. I also refrained from using the additional older brother odds ratio (OBOR) statistic. This statistic was used in some previous studies as a way of quantifying the FBOE; however, it was advocated against by Vilsmeier et al. (2023) and Raymond et al. (2025) for several reasons, including its tendency to inflate the rate of false positive results.

Additionally, another important characteristic when studying sibship effects is the participant’s sex ratio of older and younger siblings. A typical human sex ratio at birth is 106 (106 males per 100 females, corresponding to a proportion of 0.515) for white populations (Chahnazarian, 1988). A significant distortion to this ratio in a research sample could indicate the presence of a stopping rule (i.e., when parents decide to cease reproduction after having at least one child of a certain sex, at least one child of each sex, etc.; Blanchard & Skorska, 2022). If the heterosexual groups are to constitute good referential categories here, it is important that they do not deviate from the general population in their sibling sex ratios, as it could interfere with interpreting the sibship effects. To test whether the sibling sex ratios significantly depart from 106 at *p* < .05, I used a test for one proportion available online: https://www.medcalc.org/calc/test_one_proportion.php.

## Results

Descriptive statistics of the sample are summarized in Table 1. Heterosexual women were significantly older than bisexual women (*d* = 0.63). There were no significant age differences among the male groups and no differences in the mother’s age at participant’s birth for men or women. Heterosexual men reported higher importance of religious faith than bisexual men (*d* = 0.22), and heterosexual women reported higher importance of religious faith than bisexual (*d* = 0.35) and asexual (*d* = 0.57) women. Complete test results for sociodemographic variables are reported in the Supplementary Material.

Descriptive statistics on the mean number of siblings are reported in Table 3, and sibling sex ratios for each sexual orientation group are reported in Table 4. None of the ratios differed statistically significantly from the expected value of 106. Based on the results of both parameterizations, Table 5 features the effects of theoretically relevant sibship characteristics on sexual orientation. Complete results of all logistic regression models can be found in supplementary Tables S9-S16. The complementary parameterization showed that the number of older siblings (i.e., the FBOE) increased the odds of bisexuality in men (*b* = 0.51, *p* = .004, *OR* = 1.66 [95% CI 1.18, 2.35]). The three-predictor version (Zdaniuk et al., 2025) of this analysis can be found in Table S1. No other theoretically relevant predictors were statistically significant.

**Table 3:** Descriptive statistics for number of siblings across sexual orientation groups

	Men						Women					
	Heterosexual		Bisexual		Asexual		Heterosexual		Bisexual		Asexual	
	*M*	*SD*	*M*	*SD*	*M*	*SD*	*M*	*SD*	*M*	*SD*	*M*	*SD*
Sibship size (total maternal siblings)	1.42	1.14	1.42	1.26	1.26	1.35	1.25	0.94	1.36	1.18	1.22	0.87
Maternal older siblings	0.61	0.93	0.73	0.98	0.66	1.05	0.58	0.71	0.63	0.95	0.63	0.79
Maternal younger brothers	0.43	0.71	0.34	0.66	0.28	0.70	0.35	0.64	0.34	0.77	0.31	0.54
Maternal older brothers	0.30	0.63	0.41	0.68	0.34	0.76	0.28	0.53	0.31	0.58	0.34	0.55
Maternal younger sisters	0.38	0.62	0.35	0.67	0.33	0.60	0.32	0.58	0.40	0.58	0.28	0.58
Maternal older sisters	0.31	0.63	0.33	0.62	0.31	0.54	0.30	0.54	0.31	0.69	0.28	0.52

**Table 4:** Sibling sex ratios across sexual orientation groups

	Heterosexual	Bisexual	Asexual
*Older siblings*			
Men	98	125	111
Women	95	101	122
*Younger siblings*			
Men	114	95	84
Women	111	86	111

**Table 5:** Binary logistic regression results analyzing sibship effects on sexual orientation in men and women

Parameterization	Coefficient	Predictor	*b*	*SE*	*z*	*p*	*OR* (95% CI)
*Bisexual versus heterosexual men*							
Baseline	β_1_	Sibship size (FFE-ys)	− 0.10	0.14	− 0.69	0.490	0.91 (0.69–1.19)
	β_2_	Number of older siblings (SBOE)	0.14	0.18	0.76	0.447	1.15 (0.80–1.65)
	β_3_	Number of older brothers (FSED)	0.24	0.19	1.27	0.203	1.27 (0.88–1.85)
Complementary	δ_1_	Sibship size (FFE-yb)	− 0.22	0.13	− 1.70	0.089	0.80 (0.62–1.03)
	δ_2_	Number of older siblings (FBOE)	0.51	0.17	2.92	0.004	1.66 (1.18–2.35)
*Asexual versus heterosexual men*							
Baseline	β_1_	Sibship size (FFE-ys)	− 0.20	0.25	− 0.80	0.423	0.82 (0.50–1.34)
	β_2_	Number of older siblings (SBOE)	0.23	0.32	0.72	0.469	1.26 (0.67–2.35)
	β_3_	Number of older brothers (FSED)	0.07	0.33	0.23	0.819	1.08 (0.57–2.04)
Complementary	δ_1_	Sibship size (FFE-yb)	− 0.43	0.25	− 1.71	0.086	0.65 (0.40–1.06)
	δ_2_	Number of older siblings (FBOE)	0.53	0.31	1.70	0.089	1.70 (0.92–3.14)
*Bisexual versus heterosexual women*							
Baseline	β_1_	Sibship size (FFE-ys)	0.20	0.16	1.27	0.203	1.23 (0.90–1.68)
	β_2_	Number of older siblings (SBOE)	− 0.02	0.22	− 0.09	0.931	0.98 (0.63–1.52)
	β_3_	Number of older brothers (FSED)	0.10	0.22	0.45	0.653	1.10 (0.72–1.70)
Complementary	δ_1_	Sibship size (FFE-yb)	− 0.09	0.13	− 0.72	0.472	0.91 (0.70–1.18)
	δ_2_	Number of older siblings (FBOE)	0.38	0.22	1.75	0.079	1.46 (0.96–2.22)
*Asexual versus heterosexual women*							
Baseline	β_1_	Sibship size (FFE-ys)	− 0.17	0.36	− 0.47	0.637	0.85 (0.42–1.70)
	β_2_	Number of older siblings (SBOE)	0.20	0.51	0.39	0.695	1.22 (0.45–3.33)
	β_3_	Number of older brothers (FSED)	0.28	0.46	0.60	0.549	1.32 (0.53–3.26)
Complementary	δ_1_	Sibship size (FFE-yb)	− 0.17	0.32	− 0.53	0.593	0.84 (0.45–1.58)
	δ_2_	Number of older siblings (FBOE)	0.48	0.48	1.01	0.313	1.62 (0.64–4.11)

Heterosexual orientation is a reference category in all models

Although the number of older siblings (the FBOE) did not significantly increase the odds of non-heterosexuality in other study groups, the results were in the same direction and with a relatively similar effect sizes to that obtained for bisexual men. This pertained to bisexual women (*b* = 0.38, *p* = .079, *OR* = 1.46 [95% CI 0.96, 2.22]), asexual men (*b* = 0.53, *p* = .089, *OR* = 1.70 [95% CI 0.92, 3.14]), and asexual women (*b* = 0.48, *p* = .313, *OR* = 1.62 [95% CI 0.64, 4.11]).

## Discussion

In this research, I showed that the FBOE, a preponderance of older brothers previously amply demonstrated in homosexual individuals compared to heterosexual individuals (Ablaza et al., 2022; Kabátek & Blanchard, 2025; Semenyna et al., 2023, and others), is also present in a sample of bisexual men from Czechia and Slovakia. The results did not show a statistically significant FBOE in bisexual women and asexual men and women or a significant SBOE, or the difference between the FBOE and the SBOE (i.e., the FSED) in any groups.

The FBOE in bisexual men is in line with some previous studies (Blanchard, 2022; Freudenfeld et al., 2024; Sadr-Bazzaz & Vasey, 2025), but disagrees with results of other studies (Apostolou, 2020; Kabátek & Blanchard, 2025; Zdaniuk et al., 2025). Blanchard (2023) argues research samples of bisexual men may differ according to the criteria these men use to label themselves bisexual and may include various proportions of exclusively same-sex attracted men that identify as bisexual. These differences between samples might, in turn, contribute to mixed findings on the FBOE in bisexual men. In terms of magnitude, the FBOE in bisexual men is almost identical to the FBOE in Czech homosexual men previously found using this same sample (Fořt et al., 2024, Table 16) and is non-significantly larger than the previous meta-analytical FBOE estimates from convenience (*OR* = 1.47; Blanchard, 2018a) and probability (*OR* = 1.21; Blanchard, 2018b) samples.

This effect, if corroborated in further samples and by a meta-analysis, indicates that whatever the biological mechanism (e.g., maternal immunization, fetal microchimerism, see the Introduction) behind the FBOE is, it could be shared for all same-sex attracted groups—whether exclusively (homosexual) or partially (bisexual) same-sex attracted. Speculatively, this mechanism could also be shared across different mammal taxa—as suggested by a recent article (Hernández et al., 2024), which found a FBO-like effect (i.e., a tendency for individuals with same-sex partner preference to be born to multiparous females) in male laboratory rats.

I did not record a significant FBOE in bisexual women, which is in line with all previous studies (Apostolou, 2020; Blanchard, 2022; Freudenfeld et al., 2024; Kabátek & Blanchard, 2025; Sadr-Bazzaz & Vasey, 2025; Zdaniuk et al., 2025). However, the magnitude of the FBOE for bisexual women estimated in this study is similar to or larger than the FBOE estimates found for lesbian women in recent studies (Ablaza et al., 2022; Blanchard, 2022; Kabátek & Blanchard, 2025; Zdaniuk et al., 2025).

Although I did not record a significant FBOE in asexual men and women, their effect sizes (similar to that found in bisexual men) are of potential interest. Again, none of the previous studies that rigorously inspected the FBOE together with other sibship effects (Freudenfeld et al., 2024; Kabátek & Blanchard, 2025; Zdaniuk et al., 2025) found it in asexual individuals (but see Yule et al., 2014). More studies in this area are needed. I will only note that, hypothetically, asexuality in men with older brothers could be evolutionarily meaningful—being a man that does not reproduce could, under certain conditions, carry inclusive fitness benefits for such an individual’s relatives or even for the individual himself, as was shown in models by Tureček et al. (2025).

There were no significant sibship-size effects (FFE-yb or FFE-ys) in any of the groups. As previous studies (Blanchard, 2022; Kabátek & Blanchard, 2025; Sadr-Bazzaz & Vasey, 2025; Zdaniuk et al., 2025) gained conflicting results on the presence of the FFE in asexual and bisexual individuals, no valid conclusions can be made. Furthermore, it should be pointed out that Ablaza et al.’s (2022) method (used as the baseline parameterization here) may not be very well suited for studying the FFE—in formal simulations by Raymond et al. (2025), it was shown to have the lowest power of several analytical techniques used to test the FFE. In light of these findings, any conclusions about the existence of the FFE in any group should be made with caution when based on Ablaza et al.’s (2022) parameterization.

Limitations of the presented research include especially low numbers of asexual men and women, which lowered the statistical power of some analyses. For example, regarding the FBOE, the post-hoc power analysis showed acceptable power (≥ 0.80) to detect the FBOE only among bisexual men (for a more detailed description of the input parameters for the power analysis, see Data Preparation and Analyses). The power was just slightly below the commonly accepted threshold for asexual men (0.79), but lower for bisexual women (0.68), and especially for asexual women (0.46). Also, the convenience sampling method in the online environment constitutes another limitation. Although online data collection ensures anonymity for respondents, it also limits the generalizability of findings to the offline population. On the other hand, research on sibship effects in bisexual and (especially) asexual individuals is still scarce. Thus, any study inspecting sibship effects in such understudied groups is of worth. Another potential limitation is that no specific bot-detection strategies or strategies to detect fraudulent answers (other than excluding participants with unusually high number of offspring or siblings, see exclusion criterion No. 8) were employed. Nonetheless, given the relatively narrow conceptual focus of the online survey and the fact that no financial remuneration was provided (see also Sobolewski et al., 2024) to the participants, I consider the risk of possibly fraudulent answers low.

### Conclusions

In this article, I presented results regarding the fraternal birth order effect (FBOE) and other sibship effects in a sample of bisexual and asexual men and women from Czechia and Slovakia. As there is a relative dearth of studies investigating sibship effects in bisexual and especially asexual individuals, this study provides valuable insights into the potential biological origin of non-heterosexuality in these understudied groups. The results provided support for the FBOE in bisexual men (and not in other sexual minority groups), suggesting a possibility that male bisexuality might share the same biodevelopmental origin with male homosexuality. Further studies on culturally diverse samples of individuals of various sexual orientations are needed to corroborate these findings and to help constitute a solid theoretical foundation of human sexual orientation development.

## Supplementary Information

Below is the link to the electronic supplementary material.

Supplementary file1 (DOCX 60 kb)

## Data Availability

Data associated with this research and the preregistration are available publicly at Open Science Framework: https://osf.io/ugx6t/overview.

## Footnotes

1 I do not consider this a limitation—asexual people can experience sexual desire to some extent, have sexual fantasies, and have sexual encounters with other people (Hille et al., 2020; Yule et al., 2017).
