# Supplementary material for: Fraternal Birth Order Effect and Other Sibship Effects in Bisexual and Asexual Men and Women
Source: Arch Sex Behav. 2026 Sep 22;55(7):2917–28. doi: 10.1007/s10508-026-03536-9 (PMC13748132; doi:10.1007/s10508-026-03536-9)
Supplement: Supplementary file 1 [file 10508_2026_3536_MOESM1_ESM.docx]

**SUPPLEMENTARY MATERIAL**

**Supplementary Results**

Differences in age, mother’s age at participant’s birth, and importance of religious faith were tested by one-way ANOVAs, separately for each sex. Participants with a missing value were omitted from relevant analyses. Levene’s test showed significantly (*p* < 0.05) unequal variances in the age of women and in the importance of religious faith in men and women—differences in these variables were tested by Welch’s ANOVA, and Fisher's ANOVA was utilized for the remaining analyses. There were no significant differences in the age of male participants, *F*(2, 1082) = 2.20, *p* = 0.111, but there were significant between-group differences in age of women, *F*(2, 82.45) = 32.65, *p* < 0.001. Games-Howell post-hoc tests showed that heterosexual women (*M* = 31.77, *SD* = 9.78) were significantly older than bisexual women (*M* = 26.40, *SD* = 6.20), *t*(562.67) = 8.06, *p* < 0.001, *d* = 0.63. There were no between-group differences in mother’s age at participant’s birth for men, *F*(2, 779) = 0.59, *p* = 0.557, or women, *F*(2, 528) = 0.72, *p* = 0.487. There was a significant difference in the importance of religious faith for men, *F*(2, 113.57) = 3.86, *p* = 0.024, and for women, *F*(2, 93.26) = 12.42, *p* < 0.001. Games-Howell post-hoc tests showed that bisexual men (*M* = 23.75, *SD* = 28.99) were less religious than heterosexual men (*M* = 30.93, *SD* = 33.84, *t*(253.20) = 2.74, *p* = 0.018, *d* = 0.22), and that heterosexual women (*M* = 26.93, *SD* = 30.87) were more religious than bisexual (*M* = 17.15, *SD* = 23.91, *t*(509.95) = 4.05, *p* < 0.001, *d* = 0.35) and asexual (*M* = 11.19, *SD* = 18.65, *t*(48.87) = 4.15, *p* < 0.001, *d* = 0.57) women.

Additionally, differences in the number of biological sons and daughters were tested by Welch’s one-way ANOVAs, as Levene’s test indicated significant inequality of variances of these variables. There was a significant difference in the number of sons for men, *F*(2, 184.26) = 20.15, *p* < 0.001, and for women, *F*(2, 87.49) = 12.69, *p* < 0.001. Games-Howell post-hoc tests showed that heterosexual men (*M* = 0.37, *SD* = 0.73) had more sons than bisexual men (*M* = 0.23, *SD* = 0.52, *t*(358.86) = 3.09, *p* = 0.006, *d* = 0.21) and asexual men (*M* = 0.07, *SD* = 0.32, *t*(106.01) = 6.21, *p* < 0.001, *d* = 0.44). Also, bisexual men had more sons than asexual men (*t*(160.76) = 2.80, *p* = 0.016, *d* = 0.23). Heterosexual women (*M* = 0.37, *SD* = 0.71) had more sons than bisexual women (*M* = 0.14, *SD* = 0.40, *t*(539.67) = 5.05, *p* < 0.001, *d* = 0.40). There was a significant difference in the number of daughters for men, *F*(2, 142.38) = 3.84, *p* = 0.024, and for women, *F*(2, 86.98) = 12.31, *p* < 0.001. Games-Howell post-hoc tests showed that heterosexual men (*M* = 0.35, *SD* = 0.70) had more daughters than asexual men (*M* = 0.14, *SD* = 0.58, *t*(69.27) = 2.63, *p* = 0.028, *d* = 0.30). Heterosexual women (*M* = 0.37, *SD* = 0.70) had more daughters than bisexual women (*M* = 0.13, *SD* = 0.46, *t*(567.72) = 4.97, *p* < 0.001, *d* = 0.40).

Chi-squared tests indicated a significant difference in the highest level of education attained for men (χ^2^(10, *N* = 1080) = 32.75, *p* < 0.001) and women (χ^2^ (10, *N* = 609) = 57.86, *p* < 0.001), and in the population size of the childhood place of residence for men (χ^2^(8, *N* = 1081) = 18.53, *p* = 0.018). There were no significant differences in the population size of the childhood place of residence for women (χ^2^(8, *N* = 609) = 5.26, *p* = 0.729) or in the population size of the current place of residence for men (χ^2^(8, *N* = 1061) = 11.51, *p* = 0.175) or women (χ^2^(8, *N* = 590) = 6.02, *p* = 0.645).

As an additional approach to the four-predictor models presented in the full text, I replicated all analyses using a pair of logistic regressions described in Zdaniuk et al. (2025) and advocated to be used in Blanchard et al. (2025). The main difference between these models and those presented in the full text is that they lack the fourth predictor, i.e., the number of younger sisters or younger brothers, respectively. For details, see the full text and Zdaniuk et al. (2025). Results of these analyses are presented in Tables S1-S8. In terms of any theoretically relevant predictor (FBOE, SBOE, FSED, and FFE) crossing the significance threshold, the results of these additional analyses are the same as the four-predictor models presented in the full text.

**References**

Blanchard, R., Kabátek, J., & Zdaniuk, B. (2025). A current approach to logistic regression analysis of birth order and sexual orientation. *Archives of Sexual Behavior*, *54*(10), 4013–4022. https://doi.org/10.1007/s10508-025-03275-3

Zdaniuk, B., Milani, S., Makarenko, B., Marriott, N., Bogaert, A. F., & Brotto, L. A. (2025). Asexuality: Its relationship to sibling sex composition and birth order. *Archives of Sexual Behavior*, *54*(1), 51–64. https://doi.org/10.1007/s10508-024-03043-9

| Table S1: Binary logistic regression comparing sibship composition between bisexual and heterosexual men according to Zdaniuk et al.’s (2025) first model | | | | | | | |
| --- | --- | --- | --- | --- | --- | --- | --- |
|  |  |  |  |  |  | 95% CI | |
|  | *b* | *SE* | *Z* | *p* | *OR* | Lower | Upper |
| Intercept | -0.31 | 0.66 | -0.47 | 0.638 | 0.73 | 0.20 | 2.67 |
| Sibship size (FFE) | -0.17 | 0.10 | -1.68 | 0.093 | 0.85 | 0.70 | 1.03 |
| Number of older brothers (FBOE) | 0.45 | 0.15 | 2.97 | 0.003 | 1.57 | 1.17 | 2.12 |
| Number of older sisters (SBOE) | 0.21 | 0.16 | 1.31 | 0.189 | 1.23 | 0.90 | 1.67 |
| Age | 0.00 | 0.01 | 0.47 | 0.641 | 1.00 | 0.99 | 1.02 |
| Mother’s age at target’s birth | -0.05 | 0.02 | -2.23 | 0.026 | 0.95 | 0.91 | 0.99 |
| Note: Heterosexual orientation is a reference category. | | | | | | | |

| Table S2: Binary logistic regression comparing sibship composition between bisexual and heterosexual men according to Zdaniuk et al.’s (2025) second model | | | | | | | |
| --- | --- | --- | --- | --- | --- | --- | --- |
|  |  |  |  |  |  | 95% CI | |
|  | *b* | *SE* | *Z* | *p* | *OR* | Lower | Upper |
| Intercept | -0.31 | 0.66 | -0.47 | 0.638 | 0.73 | 0.20 | 2.67 |
| Sibship size (FFE) | -0.17 | 0.10 | -1.68 | 0.093 | 0.85 | 0.70 | 1.03 |
| Number of older siblings (SBOE) | 0.21 | 0.16 | 1.31 | 0.189 | 1.23 | 0.90 | 1.67 |
| Number of older brothers (FSED) | 0.25 | 0.19 | 1.29 | 0.196 | 1.28 | 0.88 | 1.86 |
| Age | 0.00 | 0.01 | 0.47 | 0.641 | 1.00 | 0.99 | 1.02 |
| Mother’s age at target’s birth | -0.05 | 0.02 | -2.23 | 0.026 | 0.95 | 0.91 | 0.99 |
| Note: Heterosexual orientation is a reference category. | | | | | | | |

| Table S3: Binary logistic regression comparing sibship composition between asexual and heterosexual men according to Zdaniuk et al.’s (2025) first model | | | | | | | |
| --- | --- | --- | --- | --- | --- | --- | --- |
|  |  |  |  |  |  | 95% CI | |
|  | *b* | *SE* | *Z* | *p* | *OR* | Lower | Upper |
| Intercept | 0.06 | 1.12 | 0.05 | 0.960 | 1.06 | 0.12 | 9.47 |
| Sibship size (FFE) | -0.33 | 0.19 | -1.74 | 0.081 | 0.72 | 0.50 | 1.04 |
| Number of older brothers (FBOE) | 0.43 | 0.27 | 1.61 | 0.108 | 1.54 | 0.91 | 2.60 |
| Number of older sisters (SBOE) | 0.35 | 0.27 | 1.28 | 0.201 | 1.42 | 0.83 | 2.42 |
| Age | -0.03 | 0.01 | -2.03 | 0.043 | 0.97 | 0.95 | 1.00 |
| Mother’s age at target’s birth | -0.06 | 0.04 | -1.71 | 0.087 | 0.94 | 0.87 | 1.01 |
| Note: Heterosexual orientation is a reference category. | | | | | | | |

| Table S4: Binary logistic regression comparing sibship composition between asexual and heterosexual men according to Zdaniuk et al.’s (2025) second model | | | | | | | |
| --- | --- | --- | --- | --- | --- | --- | --- |
|  |  |  |  |  |  | 95% CI | |
|  | *b* | *SE* | *Z* | *p* | *OR* | Lower | Upper |
| Intercept | 0.06 | 1.12 | 0.05 | 0.960 | 1.06 | 0.12 | 9.47 |
| Sibship size (FFE) | -0.33 | 0.19 | -1.74 | 0.081 | 0.72 | 0.50 | 1.04 |
| Number of older siblings (SBOE) | 0.35 | 0.27 | 1.28 | 0.201 | 1.42 | 0.83 | 2.42 |
| Number of older brothers (FSED) | 0.08 | 0.32 | 0.25 | 0.799 | 1.09 | 0.58 | 2.05 |
| Age | -0.03 | 0.01 | -2.03 | 0.043 | 0.97 | 0.95 | 1.00 |
| Mother’s age at target’s birth | -0.06 | 0.04 | -1.71 | 0.087 | 0.94 | 0.87 | 1.01 |
| Note: Heterosexual orientation is a reference category. | | | | | | | |

| Table S5: Binary logistic regression comparing sibship composition between bisexual and heterosexual women according to Zdaniuk et al.’s (2025) first model | | | | | | | |
| --- | --- | --- | --- | --- | --- | --- | --- |
|  |  |  |  |  |  | 95% CI | |
|  | *b* | *SE* | *Z* | *p* | *OR* | Lower | Upper |
| Intercept | 3.77 | 0.76 | 4.93 | < .001 | 43.26 | 9.68 | 193.29 |
| Sibship size (FFE) | 0.02 | 0.11 | 0.22 | 0.828 | 1.02 | 0.83 | 1.26 |
| Number of older brothers (FBOE) | 0.24 | 0.20 | 1.24 | 0.215 | 1.27 | 0.87 | 1.87 |
| Number of older sisters (SBOE) | 0.16 | 0.19 | 0.88 | 0.379 | 1.18 | 0.82 | 1.70 |
| Age | -0.09 | 0.01 | -7.04 | < .001 | 0.91 | 0.89 | 0.94 |
| Mother’s age at target’s birth | -0.06 | 0.02 | -2.70 | 0.007 | 0.94 | 0.90 | 0.98 |
| Note: Heterosexual orientation is a reference category. | | | | | | | |

| Table S6: Binary logistic regression comparing sibship composition between bisexual and heterosexual women according to Zdaniuk et al.’s (2025) second model | | | | | | | |
| --- | --- | --- | --- | --- | --- | --- | --- |
|  |  |  |  |  |  | 95% CI | |
|  | *b* | *SE* | *Z* | *p* | *OR* | Lower | Upper |
| Intercept | 3.77 | 0.76 | 4.93 | < .001 | 43.26 | 9.68 | 193.29 |
| Sibship size (FFE) | 0.02 | 0.11 | 0.22 | 0.828 | 1.02 | 0.83 | 1.26 |
| Number of older siblings (SBOE) | 0.16 | 0.19 | 0.88 | 0.379 | 1.18 | 0.82 | 1.70 |
| Number of older brothers (FSED) | 0.08 | 0.22 | 0.36 | 0.719 | 1.08 | 0.71 | 1.66 |
| Age | -0.09 | 0.01 | -7.04 | < .001 | 0.91 | 0.89 | 0.94 |
| Mother’s age at target’s birth | -0.06 | 0.02 | -2.70 | 0.007 | 0.94 | 0.90 | 0.98 |
| Note: Heterosexual orientation is a reference category. | | | | | | | |

| Table S7: Binary logistic regression comparing sibship composition between asexual and heterosexual women according to Zdaniuk et al.’s (2025) first model | | | | | | | |
| --- | --- | --- | --- | --- | --- | --- | --- |
|  |  |  |  |  |  | 95% CI | |
|  | *b* | *SE* | *Z* | *p* | *OR* | Lower | Upper |
| Intercept | -0.28 | 1.53 | -0.18 | 0.854 | 0.76 | 0.04 | 15.05 |
| Sibship size (FFE) | -0.17 | 0.25 | -0.68 | 0.495 | 0.84 | 0.52 | 1.37 |
| Number of older brothers (FBOE) | 0.48 | 0.42 | 1.14 | 0.253 | 1.61 | 0.71 | 3.66 |
| Number of older sisters (SBOE) | 0.20 | 0.43 | 0.47 | 0.636 | 1.22 | 0.53 | 2.83 |
| Age | -0.02 | 0.02 | -1.05 | 0.295 | 0.98 | 0.94 | 1.02 |
| Mother’s age at target’s birth | -0.05 | 0.05 | -1.04 | 0.298 | 0.95 | 0.86 | 1.05 |
| Note: Heterosexual orientation is a reference category. | | | | | | | |

| Table S8: Binary logistic regression comparing sibship composition between asexual and heterosexual women according to Zdaniuk et al.’s (2025) second model | | | | | | | |
| --- | --- | --- | --- | --- | --- | --- | --- |
|  |  |  |  |  |  | 95% CI | |
|  | *b* | *SE* | *Z* | *p* | *OR* | Lower | Upper |
| Intercept | -0.28 | 1.53 | -0.18 | 0.854 | 0.76 | 0.04 | 15.05 |
| Sibship size (FFE) | -0.17 | 0.25 | -0.68 | 0.495 | 0.84 | 0.52 | 1.37 |
| Number of older siblings (SBOE) | 0.20 | 0.43 | 0.47 | 0.636 | 1.22 | 0.53 | 2.83 |
| Number of older brothers (FSED) | 0.28 | 0.46 | 0.60 | 0.547 | 1.32 | 0.54 | 3.24 |
| Age | -0.02 | 0.02 | -1.05 | 0.295 | 0.98 | 0.94 | 1.02 |
| Mother’s age at target’s birth | -0.05 | 0.05 | -1.04 | 0.298 | 0.95 | 0.86 | 1.05 |
| Note: Heterosexual orientation is a reference category. | | | | | | | |

| Table S9: Baseline parameterization of binary logistic regression comparing sibship composition between bisexual and heterosexual men | | | | | | | |
| --- | --- | --- | --- | --- | --- | --- | --- |
|  |  |  |  |  |  | 95% CI | |
|  | *b* | *SE* | *Z* | *p* | *OR* | Lower | Upper |
| Intercept | -0.32 | 0.66 | -0.49 | 0.625 | 0.72 | 0.20 | 2.64 |
| Sibship size (FFE-ys) | -0.10 | 0.14 | -0.69 | 0.490 | 0.91 | 0.69 | 1.19 |
| Number of older siblings (SBOE) | 0.14 | 0.18 | 0.76 | 0.447 | 1.15 | 0.80 | 1.65 |
| Number of older brothers (FSED) | 0.24 | 0.19 | 1.27 | 0.203 | 1.27 | 0.88 | 1.85 |
| Number of younger brothers | -0.13 | 0.19 | -0.69 | 0.489 | 0.88 | 0.61 | 1.27 |
| Age | 0.00 | 0.01 | 0.45 | 0.653 | 1.00 | 0.99 | 1.02 |
| Mother’s age at target’s birth | -0.05 | 0.02 | -2.21 | 0.027 | 0.95 | 0.91 | 0.99 |
| Note: Heterosexual orientation is a reference category. | | | | | | | |

| Table S10: Complementary parameterization of binary logistic regression comparing sibship composition between bisexual and heterosexual men | | | | | | | |
| --- | --- | --- | --- | --- | --- | --- | --- |
|  |  |  |  |  |  | 95% CI | |
|  | *b* | *SE* | *Z* | *p* | *OR* | Lower | Upper |
| Intercept | -0.32 | 0.66 | -0.49 | 0.625 | 0.72 | 0.20 | 2.64 |
| Sibship size (FFE-yb) | -0.22 | 0.13 | -1.70 | 0.089 | 0.80 | 0.62 | 1.03 |
| Number of older siblings (FBOE) | 0.51 | 0.17 | 2.92 | 0.004 | 1.66 | 1.18 | 2.35 |
| Number of older sisters | -0.24 | 0.19 | -1.27 | 0.203 | 0.78 | 0.54 | 1.14 |
| Number of younger sisters | 0.13 | 0.19 | 0.69 | 0.489 | 1.14 | 0.79 | 1.63 |
| Age | 0.00 | 0.01 | 0.45 | 0.653 | 1.00 | 0.99 | 1.02 |
| Mother’s age at target’s birth | -0.05 | 0.02 | -2.21 | 0.027 | 0.95 | 0.91 | 0.99 |
| Note: Heterosexual orientation is a reference category. | | | | | | | |

| Table S11: Baseline parameterization of binary logistic regression comparing sibship composition between asexual and heterosexual men | | | | | | | |
| --- | --- | --- | --- | --- | --- | --- | --- |
|  |  |  |  |  |  | 95% CI | |
|  | *b* | *SE* | *Z* | *p* | *OR* | Lower | Upper |
| Intercept | 0.02 | 1.12 | 0.02 | 0.987 | 1.02 | 0.11 | 9.11 |
| Sibship size (FFE-ys) | -0.20 | 0.25 | -0.80 | 0.423 | 0.82 | 0.50 | 1.34 |
| Number of older siblings (SBOE) | 0.23 | 0.32 | 0.72 | 0.469 | 1.26 | 0.67 | 2.35 |
| Number of older brothers (FSED) | 0.07 | 0.33 | 0.23 | 0.819 | 1.08 | 0.57 | 2.04 |
| Number of younger brothers | -0.23 | 0.34 | -0.67 | 0.500 | 0.80 | 0.41 | 1.54 |
| Age | -0.03 | 0.01 | -2.04 | 0.041 | 0.97 | 0.95 | 1.00 |
| Mother’s age at target’s birth | -0.06 | 0.04 | -1.68 | 0.094 | 0.94 | 0.87 | 1.01 |
| Note: Heterosexual orientation is a reference category. | | | | | | | |

| Table S12: Complementary parameterization of binary logistic regression comparing sibship composition between asexual and heterosexual men | | | | | | | | |
| --- | --- | --- | --- | --- | --- | --- | --- | --- |
|  |  |  |  |  |  | 95% CI | |  |
|  | *b* | *SE* | *Z* | *p* | *OR* | Lower | Upper |  |
| Intercept | 0.02 | 1.12 | 0.02 | 0.987 | 1.02 | 0.11 | 9.11 |  |
| Sibship size (FFE-yb) | -0.43 | 0.25 | -1.71 | 0.086 | 0.65 | 0.40 | 1.06 |  |
| Number of older siblings (FBOE) | 0.53 | 0.31 | 1.70 | 0.089 | 1.70 | 0.92 | 3.14 |  |
| Number of older sisters | -0.07 | 0.33 | -0.23 | 0.819 | 0.93 | 0.49 | 1.76 |  |
| Number of younger sisters | 0.23 | 0.34 | 0.67 | 0.500 | 1.25 | 0.65 | 2.42 |  |
| Age | -0.03 | 0.01 | -2.04 | 0.041 | 0.97 | 0.95 | 1.00 |  |
| Mother’s age at target’s birth | -0.06 | 0.04 | -1.68 | 0.094 | 0.94 | 0.87 | 1.01 |  |
| Note: Heterosexual orientation is a reference category. | | | | | | | | |

| Table S13: Baseline parameterization of binary logistic regression comparing sibship composition between bisexual and heterosexual women | | | | | | | |
| --- | --- | --- | --- | --- | --- | --- | --- |
|  |  |  |  |  |  | 95% CI | |
|  | *b* | *SE* | *Z* | *p* | *OR* | Lower | Upper |
| Intercept | 3.74 | 0.77 | 4.88 | <0.001 | 41.98 | 9.35 | 188.39 |
| Sibship size (FFE-ys) | 0.20 | 0.16 | 1.27 | 0.203 | 1.23 | 0.90 | 1.68 |
| Number of older siblings (SBOE) | -0.02 | 0.22 | -0.09 | 0.931 | 0.98 | 0.63 | 1.52 |
| Number of older brothers (FSED) | 0.10 | 0.22 | 0.45 | 0.653 | 1.10 | 0.72 | 1.70 |
| Number of younger brothers | -0.30 | 0.20 | -1.50 | 0.133 | 0.74 | 0.50 | 1.10 |
| Age | -0.09 | 0.01 | -7.06 | <0.001 | 0.91 | 0.89 | 0.94 |
| Mother’s age at target’s birth | -0.06 | 0.02 | -2.67 | 0.008 | 0.94 | 0.90 | 0.98 |
| Note: Heterosexual orientation is a reference category. | | | | | | | |

| Table S14: Complementary parameterization of binary logistic regression comparing sibship composition between bisexual and heterosexual women | | | | | | | |  |
| --- | --- | --- | --- | --- | --- | --- | --- | --- |
|  |  |  |  |  |  | 95% CI | | |
|  | *b* | *SE* | *Z* | *p* | *OR* | Lower | Upper | |
| Intercept | 3.74 | 0.77 | 4.88 | <0.001 | 41.98 | 9.35 | 188.39 | |
| Sibship size (FFE-yb) | -0.09 | 0.13 | -0.72 | 0.472 | 0.91 | 0.70 | 1.18 | |
| Number of older siblings (FBOE) | 0.38 | 0.22 | 1.75 | 0.079 | 1.46 | 0.96 | 2.22 | |
| Number of older sisters | -0.10 | 0.22 | -0.45 | 0.653 | 0.91 | 0.59 | 1.39 | |
| Number of younger sisters | 0.30 | 0.20 | 1.50 | 0.133 | 1.35 | 0.91 | 1.99 | |
| Age | -0.09 | 0.01 | -7.06 | <0.001 | 0.91 | 0.89 | 0.94 | |
| Mother’s age at target’s birth | -0.06 | 0.02 | -2.67 | 0.008 | 0.94 | 0.90 | 0.98 | |
| Note: Heterosexual orientation is a reference category. | | | | | | | |  |

| Table S15: Baseline parameterization of binary logistic regression comparing sibship composition between asexual and heterosexual women | | | | | | | |
| --- | --- | --- | --- | --- | --- | --- | --- |
|  |  |  |  |  |  | 95% CI | |
|  | *b* | *SE* | *Z* | *p* | *OR* | Lower | Upper |
| Intercept | -0.28 | 1.53 | -0.18 | 0.854 | 0.76 | 0.04 | 15.08 |
| Sibship size (FFE-ys) | -0.17 | 0.36 | -0.47 | 0.637 | 0.85 | 0.42 | 1.70 |
| Number of older siblings (SBOE) | 0.20 | 0.51 | 0.39 | 0.695 | 1.22 | 0.45 | 3.33 |
| Number of older brothers (FSED) | 0.28 | 0.46 | 0.60 | 0.549 | 1.32 | 0.53 | 3.26 |
| Number of younger brothers | 0.00 | 0.46 | -0.01 | 0.995 | 1.00 | 0.41 | 2.45 |
| Age | -0.02 | 0.02 | -1.05 | 0.295 | 0.98 | 0.94 | 1.02 |
| Mother’s age at target’s birth | -0.05 | 0.05 | -1.04 | 0.298 | 0.95 | 0.86 | 1.05 |
| Note: Heterosexual orientation is a reference category. | | | | | | | |

| Table S16: Complementary parameterization of binary logistic regression comparing sibship composition between asexual and heterosexual women | | | | | | | |
| --- | --- | --- | --- | --- | --- | --- | --- |
|  |  |  |  |  |  | 95% CI | |
|  | *b* | *SE* | *Z* | *p* | *OR* | Lower | Upper |
| Intercept | -0.28 | 1.53 | -0.18 | 0.854 | 0.76 | 0.04 | 15.08 |
| Sibship size (FFE-yb) | -0.17 | 0.32 | -0.53 | 0.593 | 0.84 | 0.45 | 1.58 |
| Number of older siblings (FBOE) | 0.48 | 0.48 | 1.01 | 0.313 | 1.62 | 0.64 | 4.11 |
| Number of older sisters | -0.28 | 0.46 | -0.60 | 0.549 | 0.76 | 0.31 | 1.87 |
| Number of younger sisters | 0.00 | 0.46 | 0.01 | 0.995 | 1.00 | 0.41 | 2.46 |
| Age | -0.02 | 0.02 | -1.05 | 0.295 | 0.98 | 0.94 | 1.02 |
| Mother’s age at target’s birth | -0.05 | 0.05 | -1.04 | 0.298 | 0.95 | 0.86 | 1.05 |
| Note: Heterosexual orientation is a reference category. | | | | | | | |
